# Supplementary material for: Barrel cortex plasticity after photothrombotic stroke involves potentiating responses of pre-existing circuits but not functional remapping to new circuits
Source: Nat Commun. 2021 Jun 25;12:3972. doi: 10.1038/s41467-021-24211-8 (PMC8233353; doi:10.1038/s41467-021-24211-8)
Supplement: Supplementary file 1 — Supplementary Information [file 41467_2021_24211_MOESM1_ESM.pdf]

Supplementary Figures

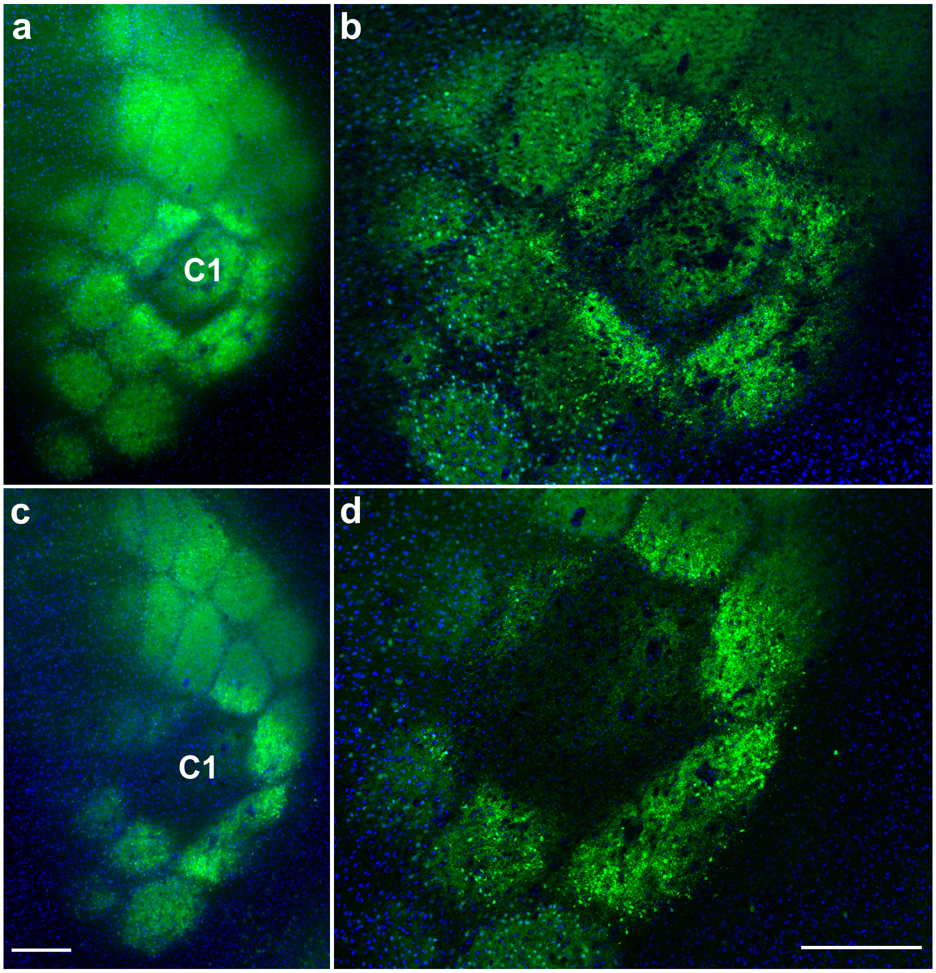

Supplementary Figure 1. Targeting of photothrombotic strokes to the C1 barrel.

A. Tangential section through layer 4 of the S1BF 5 days after photothrombotic stroke targeting the C1 barrel. Scnn1a-Tg3-Cre mice crossed with Ai162(TIT2L-GC6s-ICL-tTA2)-D mice were used to express GCaMP6s in layer 4 and highlight individual barrels in the S1BF.

B. Higher magnification of infarct area depicted in A.

C. Deeper section of layer 4 from the same mouse more clearly showing the infarct core. Scale bar in A & C = 250 μm.

D. Higher magnification of infarct area depicted in C. Note that the infarct does not extend beyond the barrels immediately surrounding the C1 barrel. Scale bar in C & D= 250 μm.

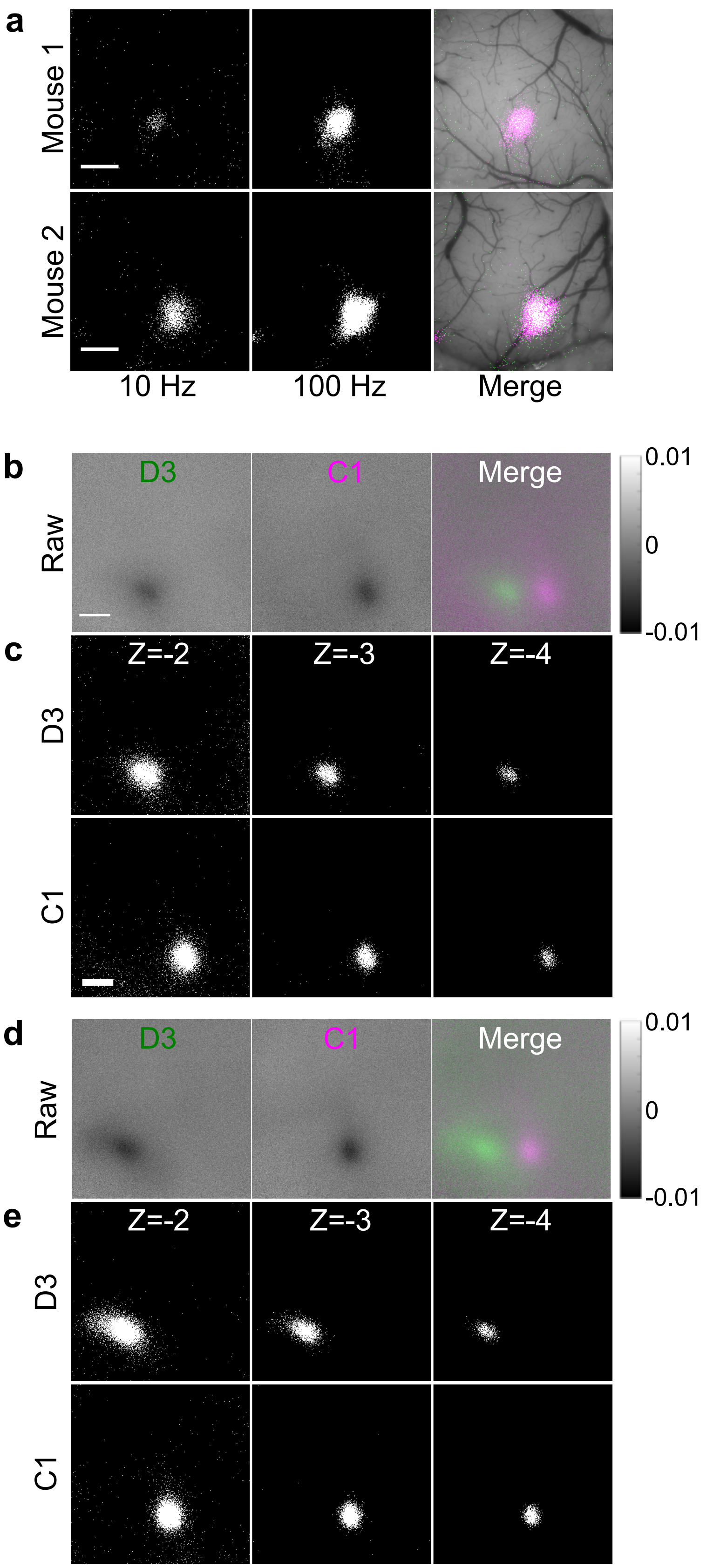
a
Mouse 1
Mouse 2
10 Hz
100 Hz
Merge
b
Raw
D3
C1
Merge
0.01
0
-0.01
c
Z=-2
Z=-3
Z=-4
D3
C1
d
Raw
D3
C1
Merge
0.01
0
-0.01
e
Z=-2
Z=-3
Z=-4
D3
C1

Supplementary Figure 2. Thresholding approach to quantify ISI whisker map area.

A. Representative data from two mice showing C1 whisker-evoked ISI maps obtained with 10 Hz (left panels) or 100 Hz (middle panels) whisker stimulation. Binary images were generated from raw ISI signals using a Z-score threshold of -2.5. Right panels: 10 Hz (green) and 100 Hz (magenta) activity maps are overlaid on photographs of the cranial window to demonstrate that both frequencies of stimulation produce maps at the same location. Scale bar = 0.5 mm.

B. Raw ISI maps elicited by stimulation of the D3 (left panel) or C1 (middle panel) whiskers in an example mouse. Right panel shows the merged D3 (green) and C1 (magenta) maps. Intensity scale indicates $\Delta R/R$ reflectance values. Note that C1 and D3 whisker-evoked ISI maps are adjacent to each other but distinct. Scale bar in B & D = 0.5 mm.

C. Binary images of D3 (top row) or C1 (bottom row) ISI maps generated from the raw ISI signals using the indicated Z-score thresholds. A z-score threshold of 'Z= -3' was chosen for quantitative analysis of map area. Scale bar in C & E= 0.5 mm.

D,E. Same as B and C, but from another animal.

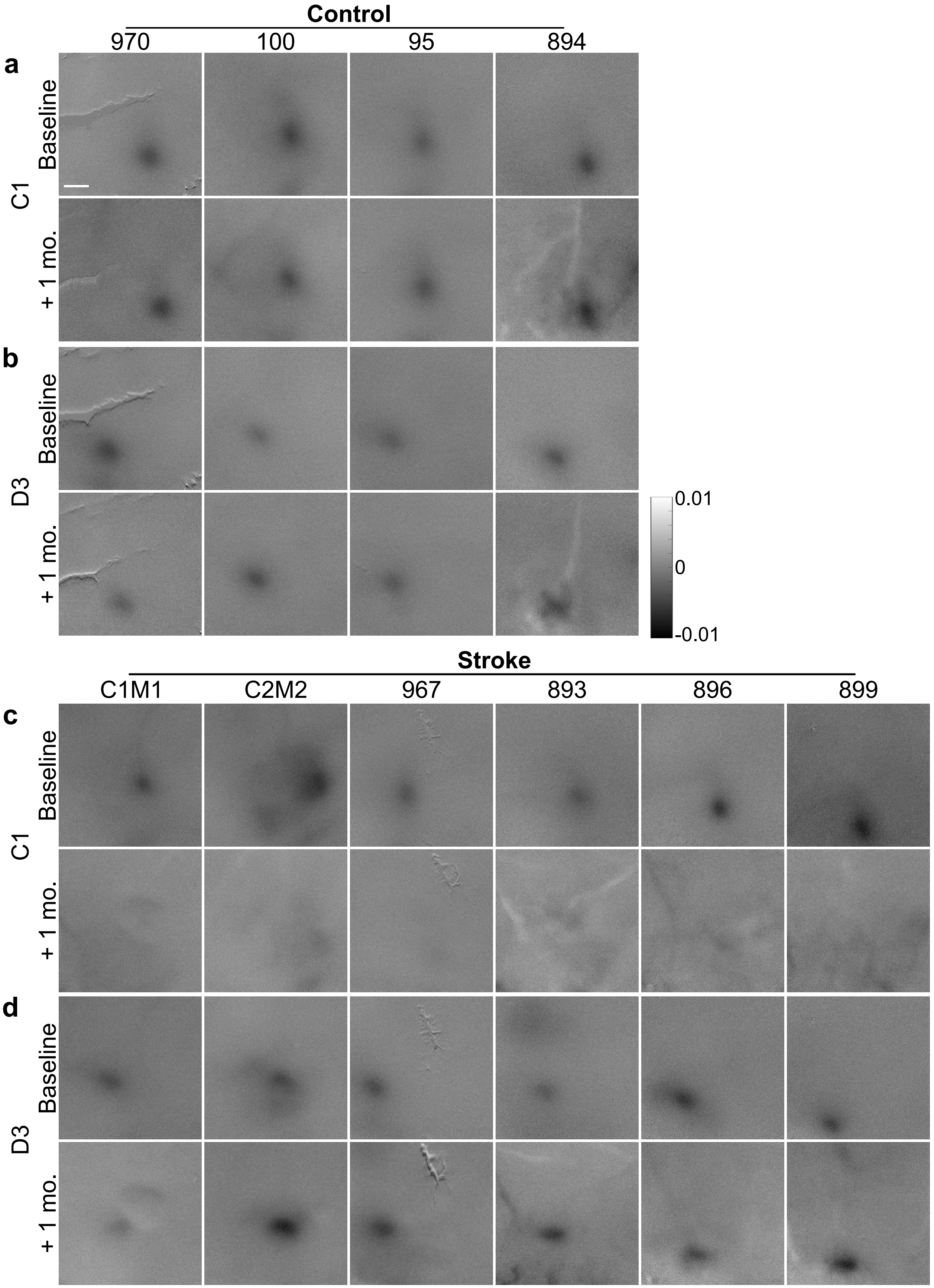

Control
970
100
95
894
a
Baseline
C1
+ 1 mo.
b
Baseline
D3
+ 1 mo.
0.01
0
-0.01
Stroke
C1M1
C2M2
967
893
896
899
c
Baseline
C1
+ 1 mo.
d
Baseline
D3
+ 1 mo.

Supplementary Figure 3. Raw ISI maps for data presented in Figure 1.

A. Raw C1 whisker-evoked ISI signals at baseline (top row) and 1 month (bottom row) after sham stroke in all control animals (n=4). Scale bar in A-D = 0.5 mm.

B. Raw D3 whisker-evoked ISI signals at baseline (top row) and 1 month (bottom row) after sham stroke in all control animals (n=4). Intensity scale indicates ΔR/R reflectance values (same for panels A-D).

C. Raw C1 whisker-evoked ISI signals at baseline (top row) and 1 month (bottom row) after C1-targeted stroke in all stroke animals (n=6).

D. Raw D3 whisker-evoked ISI signals at baseline (top row) and 1 month (bottom row) after C1-targeted stroke in all stroke animals (n=6).

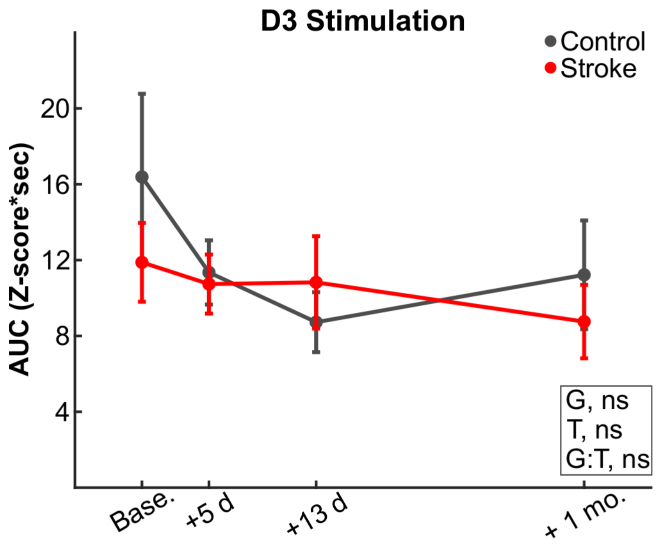

Supplementary Figure 4. D3-whisker evoked responses are stable after C1-targeted stroke.

Quantification of the AUC from the mean D3 whisker-evoked response of neurons in the D3 barrel with D3 stimulus-locked responses after stroke targeting the C1 barrel. Number of cells / number of mice for control (grey) and stroke (red) groups: baseline – 63/6 and 86/9; day 5 – 60/6 and 142/9; day 13 – 61/6 and 106/9; 1 month – 59/6 and 82/6. LME model, ANOVA for fixed effects of group (G, p=0.130), timepoint (T, p=0.208) and group-by-timepoint interaction (G:T, p=0.702). Data represent mean ± s.e.m.

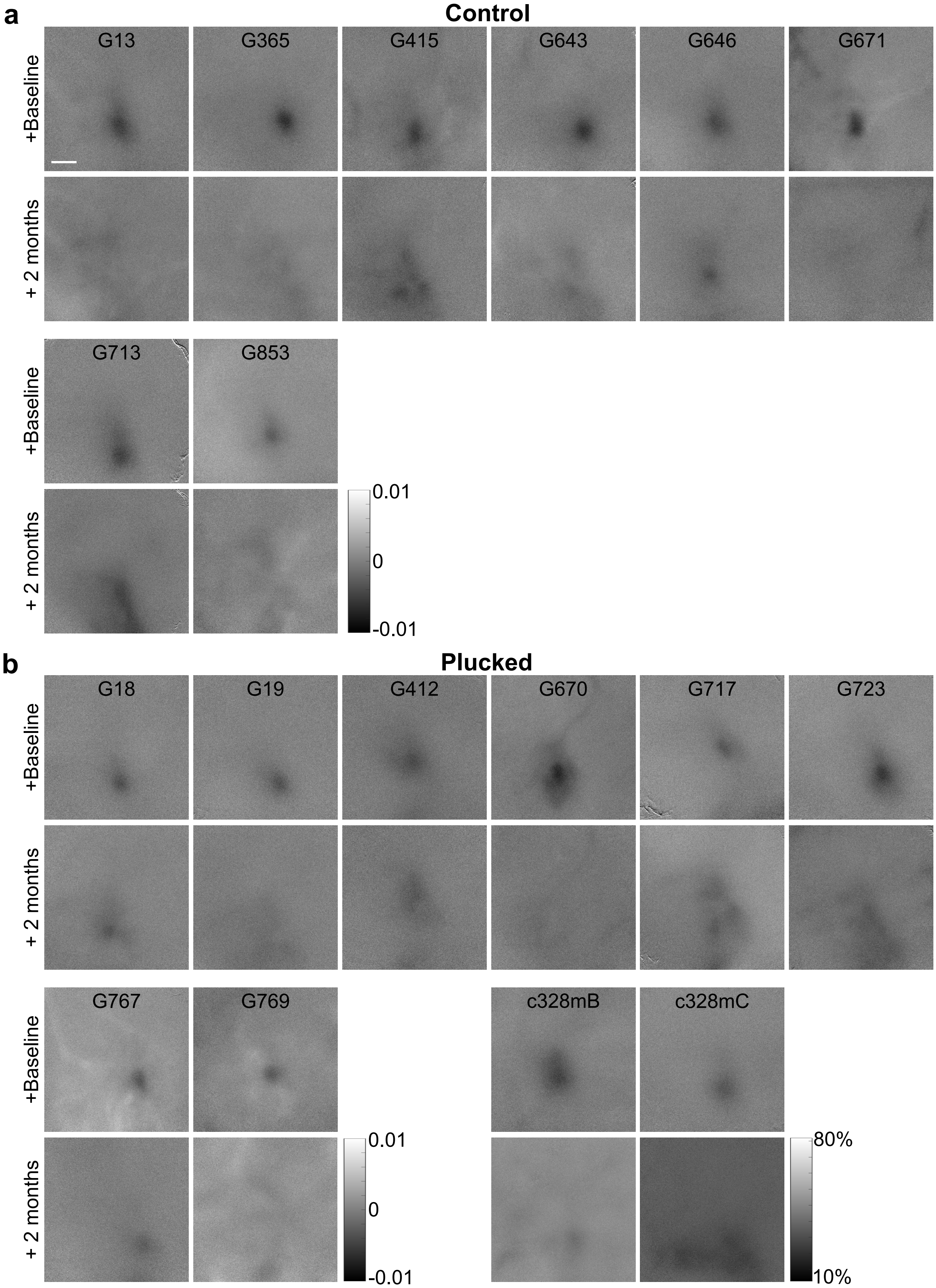
a
Control
G13
G365
G415
G643
G646
G671
+Baseline
+ 2 months
G713
G853
0.01
0
-0.01
b
Plucked
G18
G19
G412
G670
G717
G723
G767
G769
c328mB
c328mC
80%
10%

Supplementary Figure 5. Raw ISI maps for data presented in Figure 4.

A. Raw C1 whisker-evoked ISI signals at baseline (top row) and 2 months (bottom row) after C1-targeted stroke in all control animals (n=8). Intensity scale indicates ΔR/R reflectance values. Scale bar in A & B = 0.5 mm.

B. Raw C1 whisker-evoked ISI signals at baseline (top row) and 2 months (bottom row) after C1-targeted stroke in all forced use whisker plucked animals (n=10). Intensity scale indicates ΔR/R reflectance values, except for two animals (c328mB and c328mC) at 2 months post-stroke; for these two animals, the raw ΔR/R reflectance images (obtained > 2 years ago) could not be recovered, so intensity scaled (as a percentage of maximum) images were used instead.

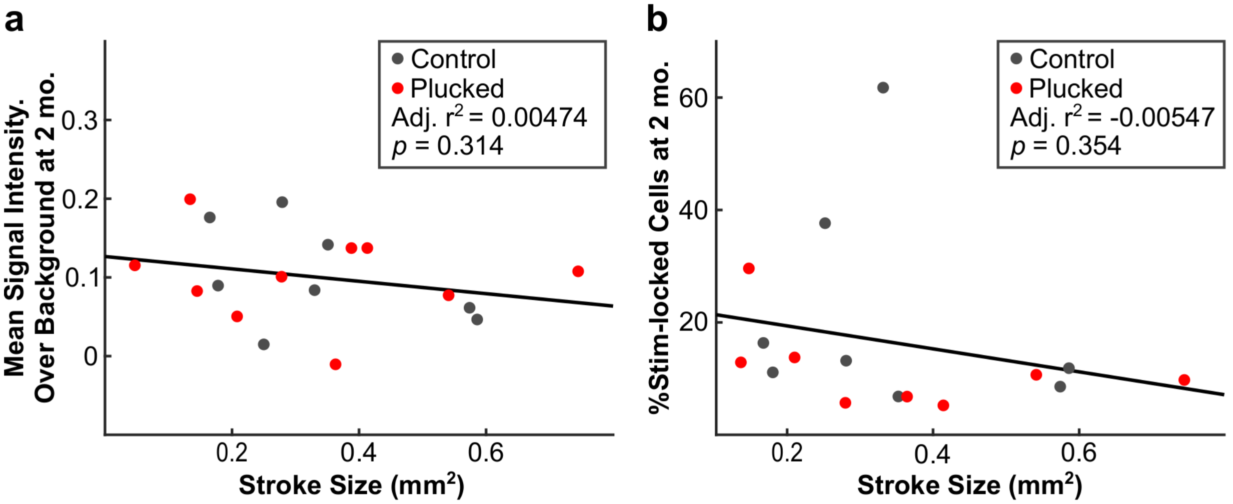

Supplementary Figure 6. No significant correlation between stroke size and C1 whisker evoked activity.

A. Correlation between C1-whisker evoked ISI signal at 2 months with stroke size at Day 5. Dots represent data from individual mice, with the plotted line representing the correlation for pooled data from both control (grey, n=8) and forced use whisker-plucked (red, n=10) groups. Linear regression model, $p=0.314$, $r^2=0.00474$.

B. Correlation between percentage of neurons with stimulus-locked responses in peri-infarct regions at 2 months with stroke size at Day 5. Dots represent data from individual mice, with the plotted line representing the correlation for pooled data from both control (grey, n=8) and plucked (red, n=8) groups. Linear regression model, $p=0.354$, $r^2=-0.00547$.

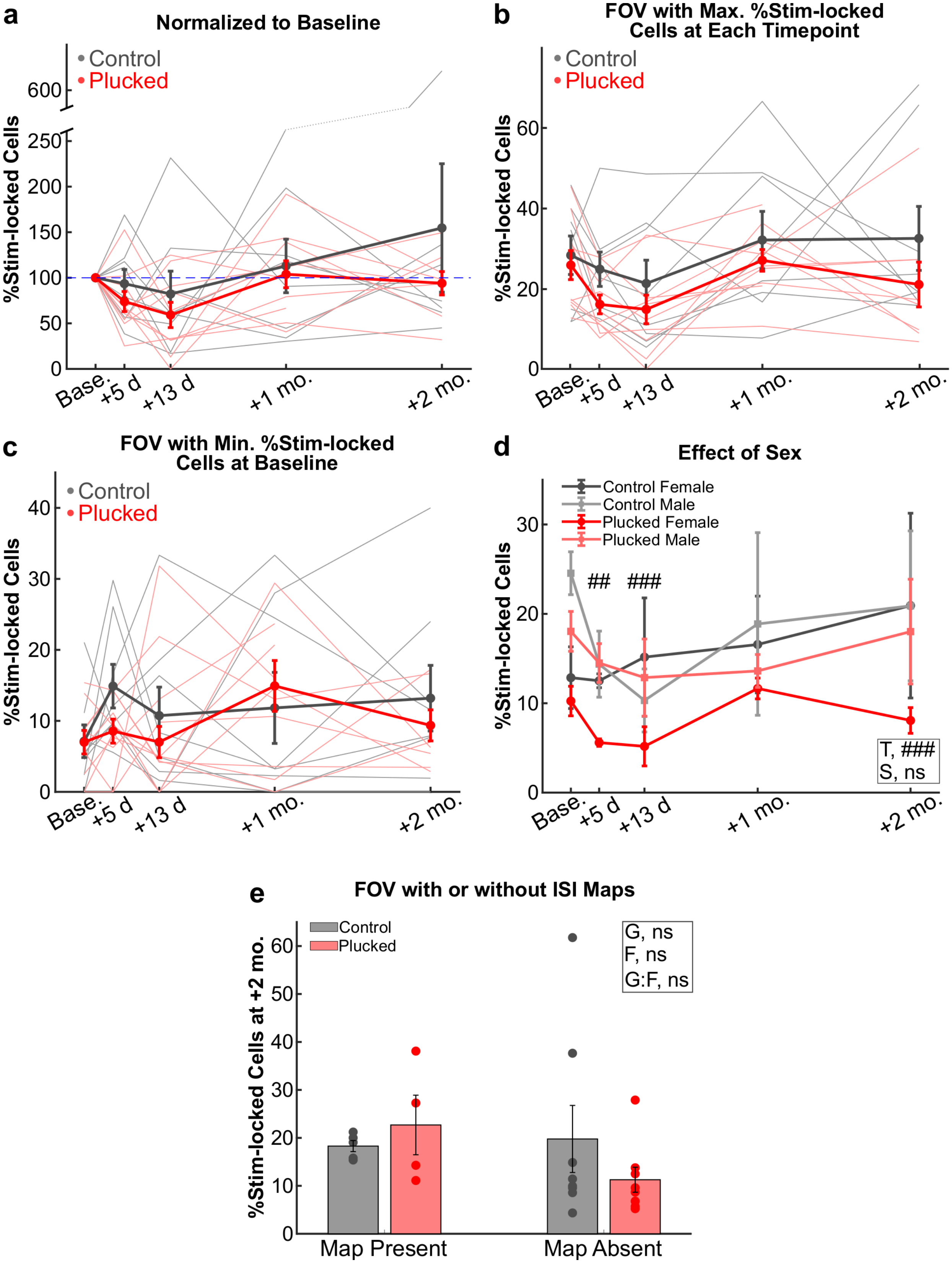

a
Normalized to Baseline
Control
Plucked
%Stim-locked Cells
Base.
+5 d
+13 d
+1 mo.
+2 mo.
b
FOV with Max. %Stim-locked Cells at Each Timepoint
Control
Plucked
%Stim-locked Cells
c
FOV with Min. %Stim-locked Cells at Baseline
Control
Plucked
%Stim-locked Cells
d
Effect of Sex
Control Female
Control Male
Plucked Female
Plucked Male
%Stim-locked Cells
##
###
T, ###
S, ns
e
FOV with or without ISI Maps
Control
Plucked
%Stim-locked Cells at +2 mo.
G, ns
F, ns
G:F, ns
Map Present
Map Absent

Supplementary Figure 7. Trends for the change in the number of C1-whisker stimulus-locked cells after stroke with forced use therapy are similar across multiple methods of analysis.

A. Percentage of neurons in peri-infarct regions across all FOV with responses that are stimulus-locked to C1-whisker stimulation normalized to the baseline percentage of stimulus-locked cells in the peri-infarct regions. N=8 and 10 mice for control (grey) and plucked (red), respectively, except n=8 for plucked group at +2 mo. Same n applies to B and C. Thick lines denote group mean, thin lines show data from individual mice. Data in A-D represent mean ± s.e.m.

B. Percentage of neurons in peri-infarct cortex with responses that are stimulus-locked to C1-whisker evoked activity when analysis is restricted to the FOV with the greatest number of stimulus-locked cells for each mouse at each timepoint.

C. Percentage of neurons in peri-infarct cortex with responses that are stimulus-locked to C1-whisker evoked activity when analysis is restricted to the FOV with the least number of stimulus-locked cells at baseline for each mouse (to test whether peri-infarct regions with the fewest stimulus-locked cells to the C1 whisker might be more capable of assuming that role post-stroke; the answer is clearly no).

D. Percentage of neurons with stimulus-locked responses to C1-whisker stimulation in the peri-infarct regions throughout recovery in control (dark grey female, light grey male) and forced use whisker plucked (dark red female, light red male) groups, sub-grouped by sex (control male, n= 3 mice; all other groups n= 5 mice, except n=3 mice for plucked male group at +2 mo). GLME binomial model, ANOVA for fixed effect of sex (S, $p= 0.080$) was not significant, whereas timepoint remained significant (T, $p<0.001$). Significance for individual coefficients for timepoints (T), corrected using Benjamini & Hochberg's method, are indicated over corresponding data points (##, $p<0.01$; ###, $p<0.001$).

E. Percentage of peri-infarct neurons with stimulus-locked responses to C1-whisker stimulation at 2 months post-stroke in control (grey) and plucked (red) groups. Imaging fields of view (FOV) with C1-whisker evoked ISI signals (“Map Present”, n=5 and 4 mice for control and plucked, respectively) were compared to FOV in areas of S1BF without detectable C1-whisker evoked ISI signal (“Map Absent”, n=8 mice for control and plucked). Two-way ANOVA, main effects of group (G, $p = 0.713$), FOV (F, $p = 0.375$), and group-by-FOV interaction (G:F, $p<0.253$) were not significant. Data represent mean ± s.e.m., with dots representing data for individual mice overlaid.

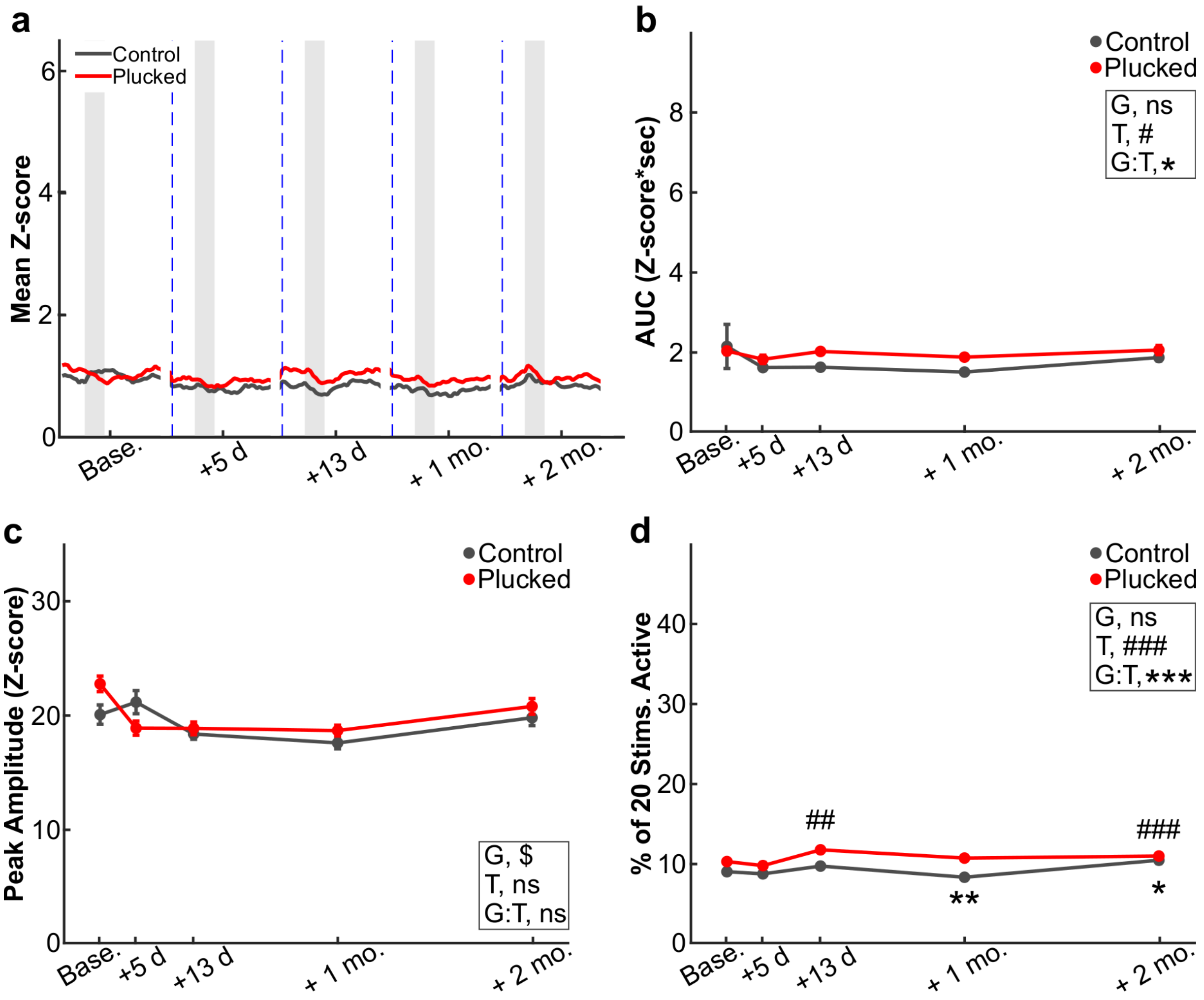

Supplementary Figure 8. Sensory-evoked responses in neurons in peri-infarct cortex that were not stimulus-locked to whisker stimulation (data plotted with same axes as in Fig. 6).

A. Mean stimulus-evoked response from non-stimulus-locked cells in control (grey) or forced use whisker-plucked groups (red) in peri-infarct regions over time following stroke. Number of cells (n) / number of mice (N) for control and plucked groups: baseline – 662/8 and 965/10; day 5 – 583/8 and 712/10; day 13 – 939/8 and 1086/10; 1 month – 733/8 and 1073/10; 2 months 637/8 and 892/8. Same n applies to B.

B. Quantification of the AUC from the mean stimulus-evoked responses in A. LME model, ANOVA for fixed effects of group (G, p=0.065), timepoint (T, #, p=0.015) and group-by-timepoint

interaction (G:T, *, p=0.021). None of the individual coefficients for timepoint (T) or group-by-timepoint interaction (G:T) were significant after correction using Benjamini & Hochberg's (B & H) method. Data in B-D represent mean ± s.e.m.

C. Quantification of peak amplitude of stimulus-evoked responses in non stimulus-locked cells over time following stroke in control (grey) and plucked (red) groups. n/N for control and plucked groups: baseline – 501/8 and 776/10; day 5 – 468/8 and 560/10; day 13 – 746/8 and 916/10; 1 month – 561/8 and 884/10; 2 months 498/8 and 720/8. LME model, ANOVA for fixed effects of group (G, $, p=0.047), timepoint (T, p=0.122) and group-by-timepoint interaction (G:T, p=0.051).

D. Quantification of fraction of whisker stimuli that neurons respond to for non-stimulus-locked cells over time following stroke in control (grey) and plucked (red) groups. n/N for control and plucked groups: baseline – 662/8 and 965/10; day 5 – 583/8 and 712/10; day 13 – 939/8 and 1086/10; 1 month – 733/8 and 1073/10; 2 months 637/8 and 892/8. GLME binomial model, ANOVA for fixed effects of group (G, p=0.137), timepoint (T, p<0.001) and group-by-timepoint interaction (G:T, p<0.001). Significance for individual coefficients for timepoint (T) or group-by-timepoint interaction (G:T), corrected using B & H method, are indicated under corresponding data points (* or #, p<0.05; ** or ##, p<0.01; *** or ###, p<0.001).

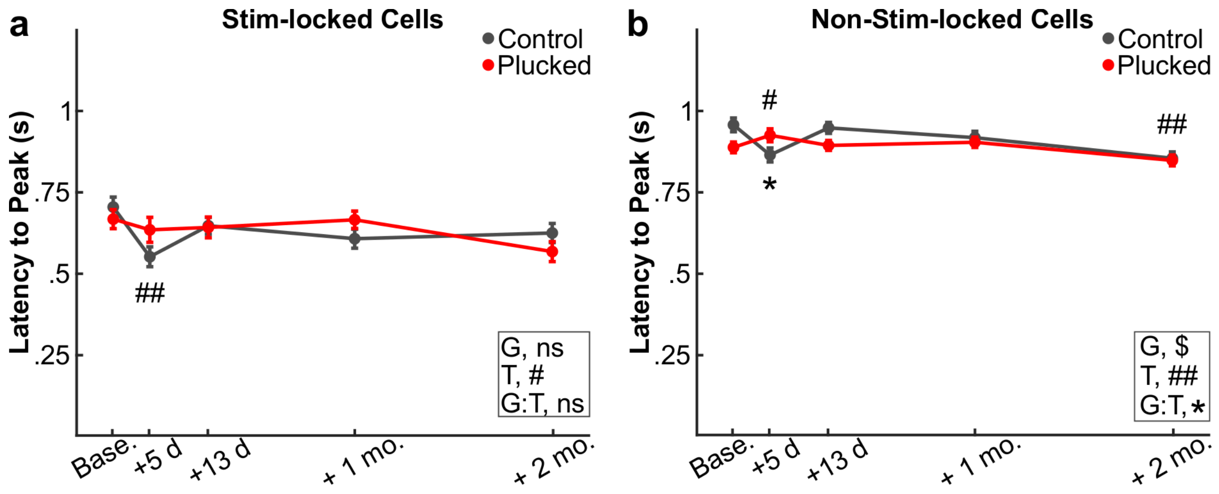

Supplementary Figure 9. Latency to sensory-evoked peak amplitude is not affected by stroke or forced use.

A. Quantification of latency to peak amplitude from whisker stimulus onset in stimulus-locked neurons over time following stroke in control (grey) and forced use whiskers plucked (red) groups. Number of cells (n)/number of mice (N) for control and plucked groups: baseline – 119/8 and 135/10; day 5 – 85/8 and 75/10; day 13 – 152/8 and 104/9; 1 month – 135/8 and 146/10; 2 months 123/8 and 112/8. LME model, ANOVA for fixed effects of group (G, *p*=0.383), timepoint (T, #, *p*=0.023) and group-by-timepoint interaction (G:T, *p*=0.144). Significance for individual coefficients for timepoint (T), corrected using Benjamini & Hochberg's (B & H) method, are indicated under corresponding data point (##, *p*<0.01). Data in A-B represent mean ± s.e.m.

B. Quantification of latency to peak amplitude from whisker stimulus onset in non-stimulus-locked neurons over time following stroke in control (grey) and plucked (red) groups. n/N for control and plucked groups: baseline – 501/8 and 776/10; day 5 – 468/8 and 560/10; day 13 – 746/8 and 916/10; 1 month – 561/8 and 884/10; 2 months 498/8 and 720/8. LME model, ANOVA for fixed effects of group (G, $, p=0.019), timepoint (T, ##, p=0.001) and group-by-timepoint interaction (G:T, *, p=0.026). Significance for individual coefficients for timepoint (T) or group-by-timepoint interaction (G:T), corrected using B & H method, are indicated under corresponding data points (* or #, p<0.05; ** or ##, p<0.01).

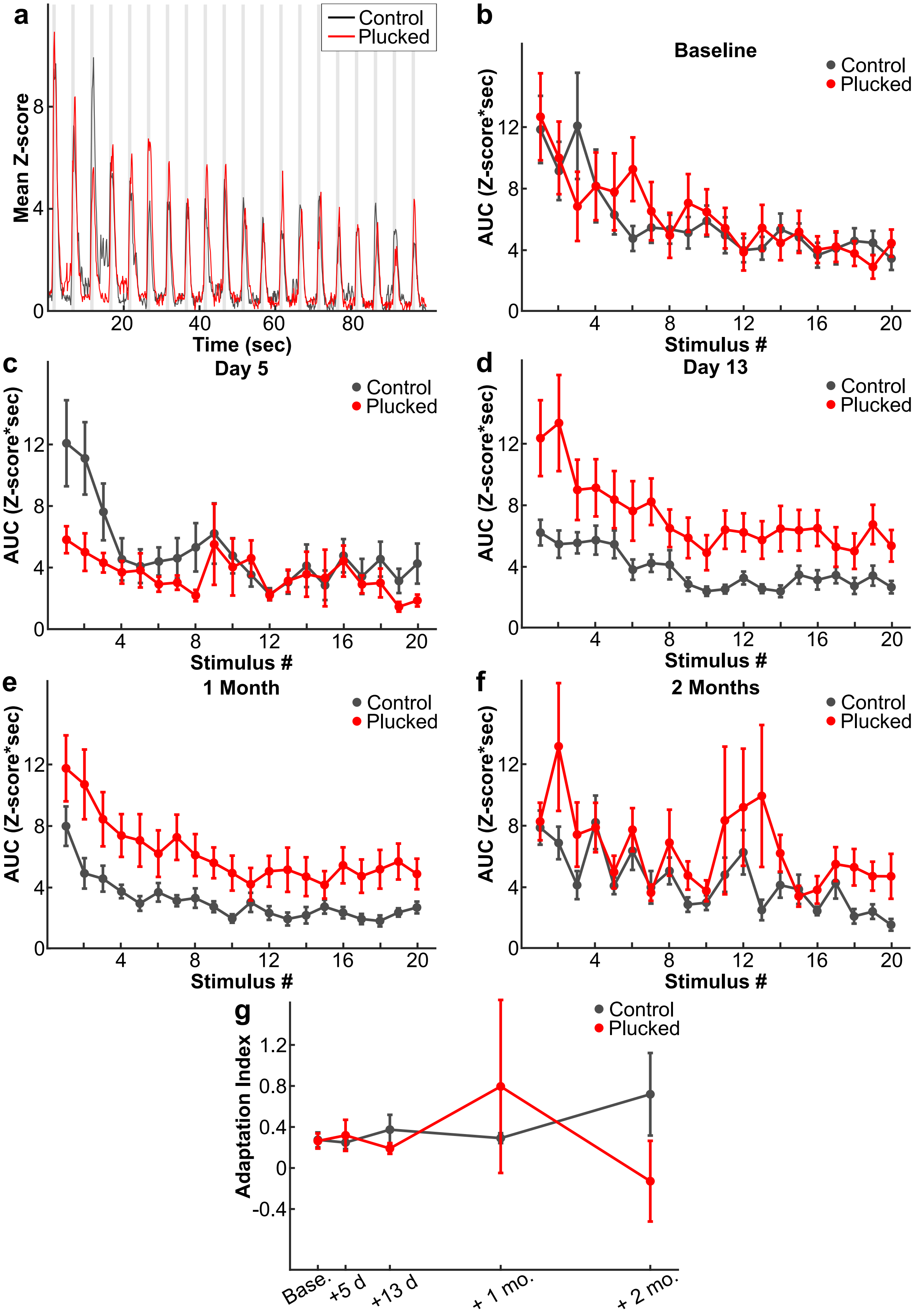
a
Control
Plucked
Mean Z-score
Time (sec)
b
Baseline
AUC (Z-score*sec)
Stimulus #
c
Day 5
d
Day 13
e
1 Month
f
2 Months
g
Adaptation Index
Base.
+5 d
+13 d
+ 1 mo.
+ 2 mo.

Supplementary Figure 10. Neuronal adaptation to repetitive whisker stimulation in peri-infarct cortex.

A. Mean Z-score of stimulus-locked cells from control (grey) or forced use whiskers plucked (red) groups at baseline, aligned to onset of whisker stimulus, for each of 20 consecutive stimuli. Number of cells / number of mice for control and plucked groups: baseline – 128/8 and 146/10; day 5 – 89/8 and 84/10; day 13 – 165/8 and 110/9; 1 month – 148/8 and 155/10; 2 months 128/8 and 119/8. Same n applies to B-G.

B-F. Quantification of mean AUC of sensory-evoked responses from control (grey) or plucked (red) groups at baseline (B), +5 days (C), +13 days (D), +1 month (E), or +2 months (F), aligned to onset of whisker stimulus, for each of 20 consecutive stimuli. Data in B-G represent mean ± s.e.m.

G. Adaptation index for sensory-evoked responses in peri-infarct cortex over time following stroke in control (grey) or plucked (red) groups. LME model, ANOVA for fixed effects of group (G, p=0.983), timepoint (T, p=0.903) and group-by-timepoint interaction (G:T, p=0.471).

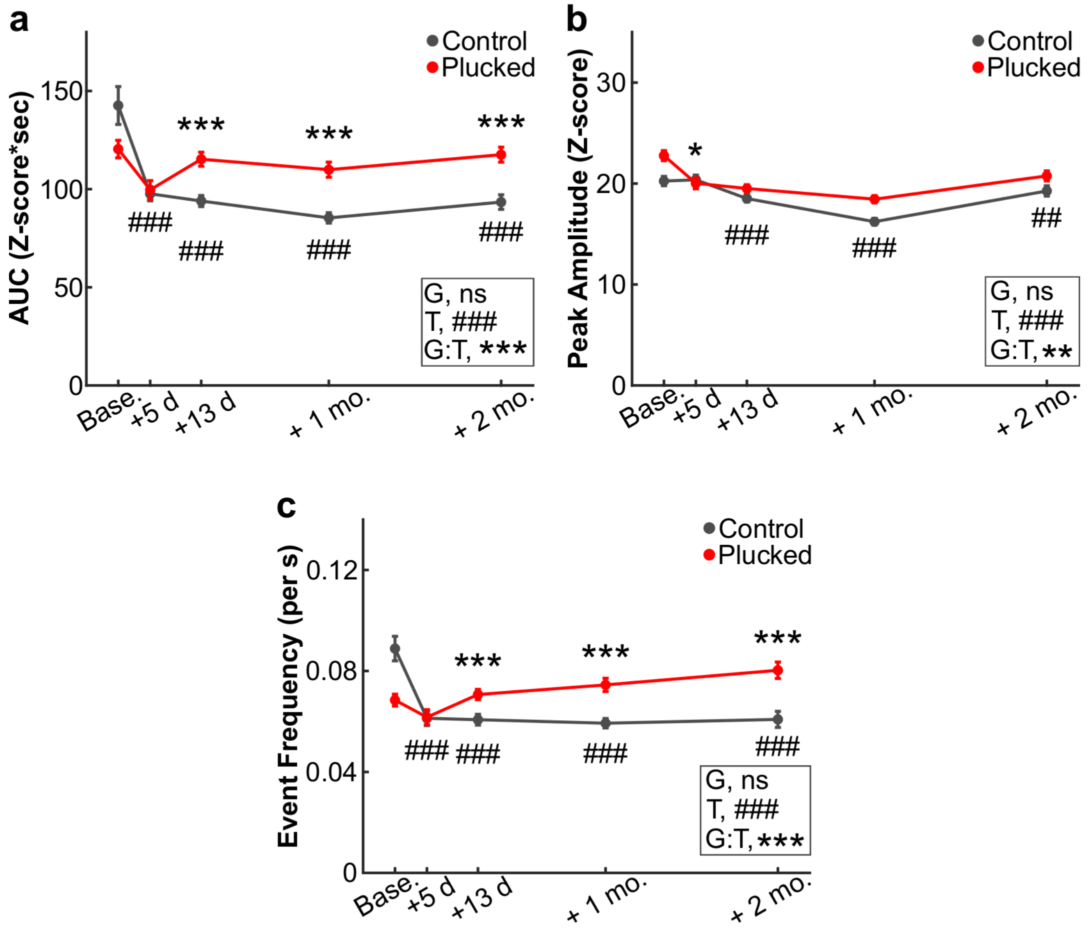

Supplementary Figure 11. Spontaneous activity of peri-infarct neurons over time before and after stroke.

A. Quantification of the AUC from the entire fluorescence trace (~100 s) of spontaneous activity from peri-infarct neurons in control (grey) and forced use whiskers plucked (red) groups before and after stroke. Number of cells (n) / number of mice (N) for control and plucked groups: baseline – 729/8 and 1045/10; day 5 – 582/8 and 636/10; day 13 – 918/8 and 1099/10; 1 month – 898/8 and 1109/10; 2 months 719/8 and 865/8. Same n/N applies to B-C. LME model, ANOVA for fixed effects of group (G, $p=0.398$), timepoint (T, ###, $p<0.001$) and group-by-timepoint interaction (G:T, ***, $p<0.001$). Significance for individual coefficients for timepoint (T) or group-by-timepoint interaction (G:T), corrected using Benjamini & Hochberg's (B & H)

method, are indicated under corresponding data points (*** or ###, $p<0.001$). Data in A-C represent mean ± s.e.m.

B. Quantification of peak amplitude of calcium events for peri-infarct neurons in control (grey) and plucked (red) groups before and after stroke. LME model, ANOVA for fixed effects of group (G, $p=0.346$), timepoint (T, ###, $p<0.001$) and group-by-timepoint interaction (G:T, **, $p=0.002$). Significance for individual coefficients for timepoint (T) or group-by-timepoint interaction (G:T), corrected using B & H method, are indicated under corresponding data points (* or #, $p<0.05$; ** or ##, $p<0.01$; *** or ###, $p<0.001$).

C. Quantification of frequency of calcium events for peri-infarct neurons in control (grey) and forced use whiskers plucked (red) groups before and after stroke. LME model, ANOVA for fixed effects of group (G, $p=0.247$), timepoint (T, ###, $p<0.001$) and group-by-timepoint interaction (G:T, ***, $p<0.001$). Significance for individual coefficients for timepoint (T) or group-by-timepoint interaction (G:T), corrected using B & H method, are indicated under corresponding data points (*** or ###, $p<0.001$).
