# Supplementary material for: Barrel cortex plasticity after photothrombotic stroke involves potentiating responses of pre-existing circuits but not functional remapping to new circuits
Source: Nat Commun. 2021 Jun 25;12:3972. doi: 10.1038/s41467-021-24211-8 (PMC8233353; doi:10.1038/s41467-021-24211-8)
Supplement: Supplementary file 2 — Reporting Summary [file 41467_2021_24211_MOESM2_ESM.pdf]

nature research

Corresponding author(s): Carlos Portera-Cailliau

Last updated by author(s): May 10, 2021

Reporting Summary

Nature Research wishes to improve the reproducibility of the work that we publish. This form provides structure for consistency and transparency in reporting. For further information on Nature Research policies, see our Editorial Policies and the Editorial Policy Checklist.

Statistics

For all statistical analyses, confirm that the following items are present in the figure legend, table legend, main text, or Methods section.

n/a	Confirmed	
☐	☒	The exact sample size (n) for each experimental group/condition, given as a discrete number and unit of measurement
☐	☒	A statement on whether measurements were taken from distinct samples or whether the same sample was measured repeatedly
☐	☒	The statistical test(s) used AND whether they are one- or two-sided<br>*Only common tests should be described solely by name; describe more complex techniques in the Methods section.*
☐	☒	A description of all covariates tested
☐	☒	A description of any assumptions or corrections, such as tests of normality and adjustment for multiple comparisons
☐	☒	A full description of the statistical parameters including central tendency (e.g. means) or other basic estimates (e.g. regression coefficient) AND variation (e.g. standard deviation) or associated estimates of uncertainty (e.g. confidence intervals)
☐	☒	For null hypothesis testing, the test statistic (e.g. F, t, r) with confidence intervals, effect sizes, degrees of freedom and P value noted<br>*Give P values as exact values whenever suitable.*
☒	☐	For Bayesian analysis, information on the choice of priors and Markov chain Monte Carlo settings
☒	☐	For hierarchical and complex designs, identification of the appropriate level for tests and full reporting of outcomes
☒	☐	Estimates of effect sizes (e.g. Cohen's d, Pearson's r), indicating how they were calculated

Our web collection on statistics for biologists contains articles on many of the points above.

Software and code

Policy information about availability of computer code

Data collection	ScanImage (version 3.8, Vidrio Technologies) was used for collection of 2P imaging data. Zen Pro v2.5 (Zeiss) was used for imaging of fixed brain sections. MATLAB (version 2009a, Mathworks) was used to control image acquisition for intrinsic signal imaging.
Data analysis	2P imaging data were analyzed using custom written MATLAB scripts as we have previously described (He, C. X. et al., J. Neurosci. 37, 6475–6487 (2017)). We also used the PeakFinder script (Mathworks File Exchange, version 2.0.2.0). Quantification of TRAP labeling as analyzed using ImageJ (v2.1.0). Intrinsic Signal Imaging data was analyzed using ImageJ and MATLAB (v2020a). All statistical analyses were performed using MATLAB (v2020a).

For manuscripts utilizing custom algorithms or software that are central to the research but not yet described in published literature, software must be made available to editors and reviewers. We strongly encourage code deposition in a community repository (e.g. GitHub). See the Nature Research guidelines for submitting code & software for further information.

Data

Policy information about availability of data

All manuscripts must include a data availability statement. This statement should provide the following information, where applicable:

- Accession codes, unique identifiers, or web links for publicly available datasets
- A list of figures that have associated raw data
- A description of any restrictions on data availability

The data generated and analyzed for this study are available from the corresponding author upon reasonable request.

Field-specific reporting

Please select the one below that is the best fit for your research. If you are not sure, read the appropriate sections before making your selection.

[x] Life sciences [ ] Behavioural & social sciences [ ] Ecological, evolutionary & environmental sciences

For a reference copy of the document with all sections, see nature.com/documents/nr-reporting-summary-flat.pdf

Life sciences study design

All studies must disclose on these points even when the disclosure is negative.

Sample size	Sample sizes were not based on a priori power calculations but are consistent with other studies in the field using similar techniques, including our own (He, C. X. et al., J. Neurosci. 37, 6475–6487 (2017); Mostany, R. et al., J. Neurosci. 30, 14116–14126 (2010); Goel, A. et al., Nat. Neurosci. 21, 1404–1411 (2018); Johnston, D. G. et al., Cereb. Cortex 23, 751–762 (2013)).
Data exclusions	Animals in which PT strokes were mistargeted were excluded from analysis. We did not use pre-established criteria, but excluded mice qualitatively as judged based on post-stroke day 5 imaging of cerebral vasculature compared to pre-stroke ISI maps. In some animals, the cranial window did not remain optically transparent for every single imaging time point. As a result, data for some time points could not be collected. For the analysis of the percentage of neurons with stimulus-locked response to C1 and D3 whisker stimulation, comparing sham versus stroke groups, the following data points were missing: one mouse in the stroke group at 13 d post-stroke and 3 mice in the stroke group at one month post-stroke. For the analysis of sensory-evoked responses in control versus forced use whisker plucked groups, one mouse in the plucked group had no neurons with stimulus-locked responses to C1 whisker stimulation at +13 d post-stroke, and 2 mice in the plucked group were not imaged at 2 months post-stroke. Data from the remaining time points for these mice were included in the analysis and the missing time points were considered "missing at random".
Replication	Our study consists of two cohorts of animals subjected to C1 barrel PT strokes. In the first we compared animals receiving sham stroke to PT stroke. In the second, we compared animals with stroke and no intervention with animals with stroke plus forced use therapy. Our main finding that C1 whisker responsive cells are not increased in peri-infarct regions after stroke, was reproduced between these two distinct cohorts.
Randomization	Animals were randomized to groups at the time of the stroke intervention. Randomization was performed by the experimenter to balance group size and gender as closely between groups as possible.
Blinding	It was not possible to blind experimenters during image acquisition because both the presence of strokes as well as the presence or absence of whiskers were readily visible. For data analysis, imaging files were randomized and blinded and processed in batches for region-of-interest selection and quantification of whisker responsivity and neuronal activity measures.

Reporting for specific materials, systems and methods

We require information from authors about some types of materials, experimental systems and methods used in many studies. Here, indicate whether each material, system or method listed is relevant to your study. If you are not sure if a list item applies to your research, read the appropriate section before selecting a response.

Materials & experimental systems

n/a	Involved in the study	
[x]	[ ]	Antibodies
[x]	[ ]	Eukaryotic cell lines
[x]	[ ]	Palaeontology and archaeology
[ ]	[x]	Animals and other organisms
[x]	[ ]	Human research participants
[x]	[ ]	Clinical data
[x]	[ ]	Dual use research of concern

Methods

n/a	Involved in the study	
[x]	[ ]	ChIP-seq
[x]	[ ]	Flow cytometry
[x]	[ ]	MRI-based neuroimaging

Animals and other organisms

Policy information about studies involving animals; ARRIVE guidelines recommended for reporting animal research

Laboratory animals	Both male and female mice were used, beginning at 6-10 weeks old at the time of cranial window surgery. All animals were housed in a vivarium with a 12 h light/dark cycle. For in vivo imaging, we used transgenic Thy1-GCaMP6s mice (GP4.3, JAX line 024275)64. For activity-dependent labeling, we used the TRAP (Targeted Recombination in Active Populations) approach41, crossing cFos-CreERT2 mice (JAX line 021882) with the Ai9 Cre-dependent tdTomato reporter line (JAX line 007909). All transgenic lines were maintained on a C57BL/J6 background.
Wild animals	The study did not involve wild animals.

Field-collected samples	The study did not involve field-collected samples.
Ethics oversight	All experiments followed the U.S. National Institutes of Health guidelines for animal research, under an animal use protocol approved by the Chancellor's Animal Research Committee (ARC) and Office for Animal Research Oversight at the University of California, Los Angeles (#2005-145).

Note that full information on the approval of the study protocol must also be provided in the manuscript.
